# Benefits of Early Treatment for Patients with Hepatic Myelopathy Secondary to TIPS: A Retrospective Study in Northern China

**DOI:** 10.1038/s41598-018-33216-1

**Published:** 2018-10-12

**Authors:** Hongwei Zhao, Zhendong Yue, Lei Wang, Zhenhua Fan, Fuliang He, Xiaoqun Dong, Fuquan Liu

**Affiliations:** 1 Department of Interventional Therapy, Beijing Shijitan Hospital, Capital Medical University, Beijing, China; 2 Department of Medicine, The Warren Alpert Medical School, Brown University, Providence, RI 02903 USA

## Abstract

Transjugular intrahepatic portosystemic shunt (TIPS) is an effective therapy for reducing portal pressure. Hepatic myelopathy (HM), a rare complication of chronic liver diseases, remains obscure in terms of treatment and prognosis. We aimed to determine an optimal treat strategy for patients with HM after TIPS. Twenty-nine patients who developed HM after TIPS were stratified by time-lapse from onset to treatment: group A (n = 16), <6 months; group B (n = 13), ≥6 months. Therapeutic measures included shunt-limiting and medical treatments. Overall survival, lower-limb muscle strength, Fugl-Meyer score, Barthel index, and serum ammonia were recorded. Median survival time in group A or B was 30 months or 16.5 months, respectively (log rank p = 0.0172). All patients in group A obtained improvement in grading of muscle strength (p < 0.0001), Fugl-Meyer score (p = 0.0021), and Barthel index (p = 0.0003), particularly male patients and those subjected to shunt-limiting. Serum ammonia levels were decreased significantly in both group A (p = 0.0007) and group B (p = 0.0007). Collectively, once HM is confirmed after TIPS, active intervention is imperative and urgent, especially within the first 6 months from onset of symptom. TIPS shunt-limiting is particularly beneficial for rehabilitation in patients with early-onset HM.

## Introduction

Upper gastrointestinal hemorrhage and refractory ascites caused by portal hypertension significantly affect patients’ daily activities, which may be life threatening. Transjugular intrahepatic portosystemic shunt (TIPS) is highly effective in reducing portal pressure. Rarely, hepatic myelopathy (HM) can be developed as a consequence of TIPS resulting from severe liver dysfunction. HM is characterized by spastic paraparesis and minimal sensory abnormalities in patients with chronic liver diseases including cirrhosis. HM is commonly manifested in conjunction with acute and chronic liver necrosis or end-stage cirrhosis, particularly stemming from surgically created or spontaneous portosystemic shunts^1^.

Unfortunately, little is known about treatment and prognosis in this setting. Not until gait impairment ensues, would patients with HM receive conservative treatments, including protein restriction and oral lactulose. However, such measures have failed to prevent progressive mobility declines, culminating in cane or wheelchair use to ensure balance and stability. Other than early liver transplantation (LT), no generally accepted effective treatment is available for HM to date^2–5^; and in patients with normal liver function or Child-Pugh A-B grade cirrhosis, the choice of LT (vs. other treatments) is debated^6,7^.

Aside from conservative medical management, we attempted to rectify consequences of original TIPS procedures by placement of reducing stents. We anticipated that this strategy might effectively improve both quality of life and symptoms in affected patients.

## Result

**Figure 1:**
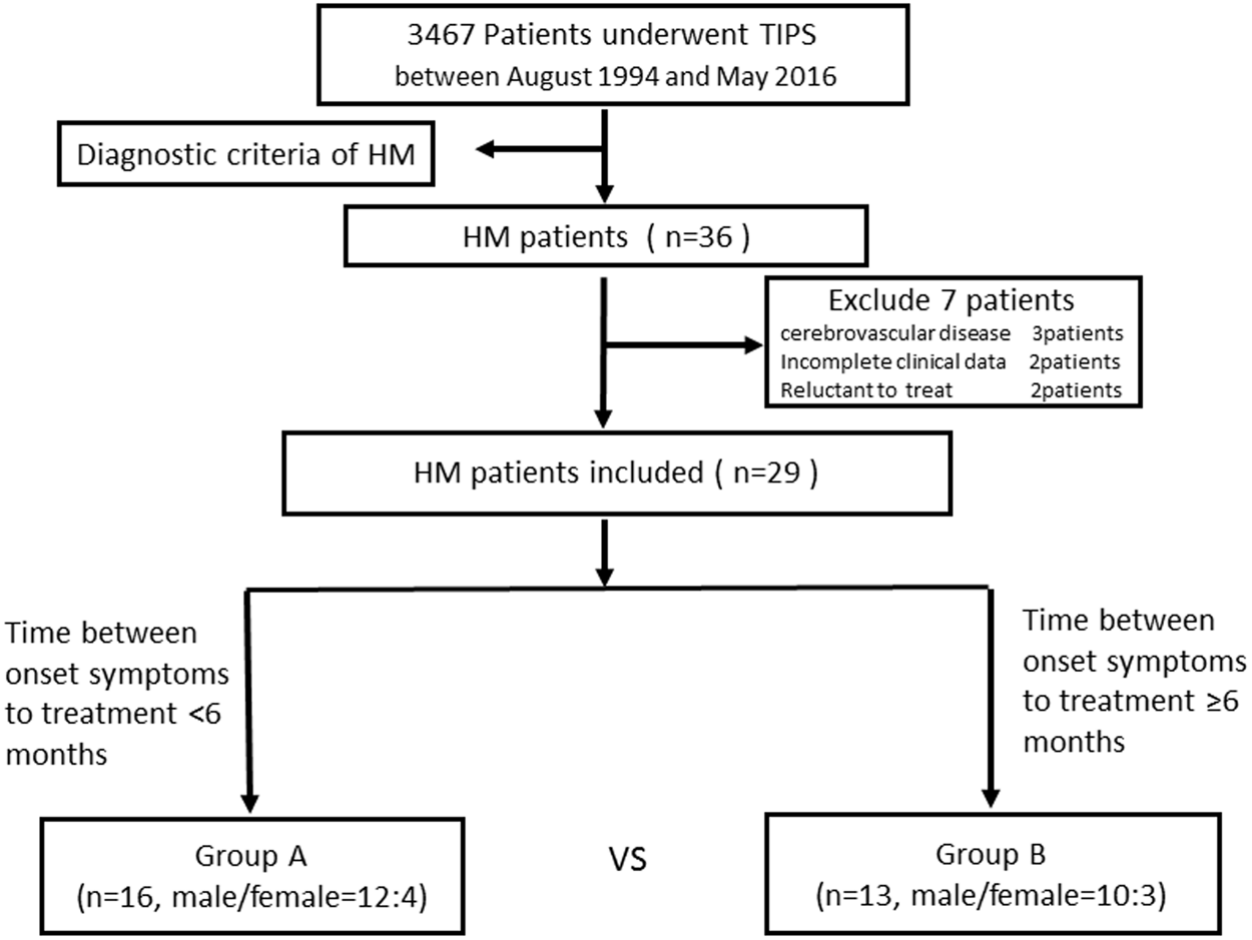
Flowchart of multi-center enrollment of HM patients who underwent TIPS between 1994 and 2016 in Northern China.

Totally 36 patients with HM were identified for this retrospective study. Patients with HM accounted for 1.04% (36/3467) of all patients undergoing TIPS during the study period. Any candidates unable to provide informed consent or complete information were excluded from analysis. In total, 29 patients (22 male and 7 female) were proved eligible for final analysis (Fig. 1). All patients displayed similar symptoms and disease evolution, initially presenting as heaviness of the lower limbs. One patient experienced urinary incontinence. Early electromyography examination was normal in 26 patients. All the patients underwent preoperative computed tomography and ultrasound examinations. Symmetrical hyperintensity was identified along lateral pyramidal (corticospinal) tracts bilaterally on regular/FLAIR MR images as a nonspecific finding. Clinical and demographic information of the study population is summarized in Table 1.

**Table 1:** The clinical and demographic information of HM patients

Index	Group A (N = 16) (n, %)	Group B (N = 13) (n, %)	T/χ^2^	P
Age (years)^*^	39.5 ± 6.3	40.2 ± 3.3	2.369	0.976
Male	12 (75%)	10 (76.92%)	0.0145	0.9042
Etiology				
Virus related cirrhosis (HBV) HCV)	10 (62.5%)	9 (69.23%)	0.1438	0.7045
Alcoholic cirrhosis	4 (25%)	3 (23.08%)	0.0145	0.9042
Budd-Chiari Syndrome	1 (6.25%)	0 (0)	0.8415	0.359
Autoimmune cirrhosis	1 (6.25%)	1 (7.69%)	0.0232	0.8788
Clinical characteristics				
Gastrointestinal hemorrhage	8 (50%)	7 (53.85%)	0.0425	0.8367
Refractory ascites	5 (31.25%)	4 (30.77%)	0.0008	0.9778
Hemorrhage + ascites	3 (18.75%)	2 (15.38%)	0.0569	0.8114
Diameter of stent (original TIPS)				
8 mm	9 (56.25%)	7 (53.85%)	0.0168	0.897
10 mm	7 (43.75%)	6 (46.15%)	0.0168	0.897
HE symptoms before TIPS	6 (37.5%)	5 (38.46%)	0.0028	0.9577
PSG before TIPS (mmHg)^*^	19.38 ± 2.80	18.31 ± 1.84		0.2468
PSG after TIPS (mmHg)^*^	8.63 ± 0.89	9.08 ± 1.04		0.2789
HVPG before original TIPS (mmHg)^*^	13.25 ± 1.81	12.25 ± 1.41		0.1213
Time from TIPS to HM symptoms (months)^*^	9.81 ± 7.09	8.31 ± 6.82		0.6577
Serum total bilirubin (mg/dL)^*^	1.76 ± 0.26	1.71 ± 0.26		0.7722
Serum albumin (g/L)^*^	30.69 ± 1.45	30.69 ± 1.25		0.8162
International normalized ratio^*^	1.11 ± 0.14	1.12 ± 0.16		0.8618
Aspartate transaminase (U/L)^*^	39.44 ± 9.44	36.46 ± 8.24		0.3793
Alanine transaminase (U/L)	65.94 ± 13.36	65.46 ± 7.89		0.4527
Serum sodium (mmol/L)^*^	138.4 ± 5.9	142.7 ± 4.63		0.0363
Serum creatinine (mg/dL)^*^	0.98 ± 0.13	0.95 ± 0.13		0.3156
MELD score^*^	9.88 ± 1.54	10.08 ± 1.66		0.3672
Child-Pugh score^*^	7.5 ± 1.41	7.62 ± 1.19		0.3853
Liver function Child-Pugh				
A	5 (31.25%)	3 (23.08%)	0.2398	0.6243
B	9 (56.25%)	9 (69.23%)	0.5133	0.4737
C	2 (38.38)	1 (7.69%)	0.1787	0.6725

^*^Data are means ± standard deviation.

### Laboratory and cranial MR imaging changes

With respect to liver function indices, only serum ALB concentrations in male patients were significantly decreased in group A (from 30.5 ± 1.17 to 29.92 ± 1.06 g/L; t = 2.461; p = 0.032). Serum ammonia levels were significantly reduced in all patients (from 76.88 ± 5.03 to 70.69 ± 3.95 mmol/L; t = 4.259; p = 0.0007), either medical therapy (from 76.29 ± 2.63 to 70.86 ± 4.38 mmol/L; t = 4.478; p = 0.0042) or stent-limiting treatment (from 77.33 ± 6.46 to 70.56 ± 3.84 mmol/L; t = 2.749; p = 0.0251) was pursued. In group B, significant decline in serum ALT was observed (from 69.77 ± 5.82 to 73.92 ± 4.86 U/L; t = 2.299; p = 0.0363). Blood ammonia levels were significantly reduced in overall patients (from 77.31 ± 9.19 to 60.38 ± 8.44 mmol/L; t = 4.549; p = 0.0007), especially male patients (from 76.7 ± 9.38 to 58.6 ± 7.86 mmol/L; t = 4.634; p = 0.0012), either medical therapy (from 78.4 ± 10.99 to 57 ± 8.89 mmol/L; t = 3.667; p = 0.0215) or stent-limiting treatment (from 76.63 ± 8.62 to 62.5 ± 7.98 mmol/L; t = 2.915; p = 0.0225) was undertaken.

**Figure 2:**
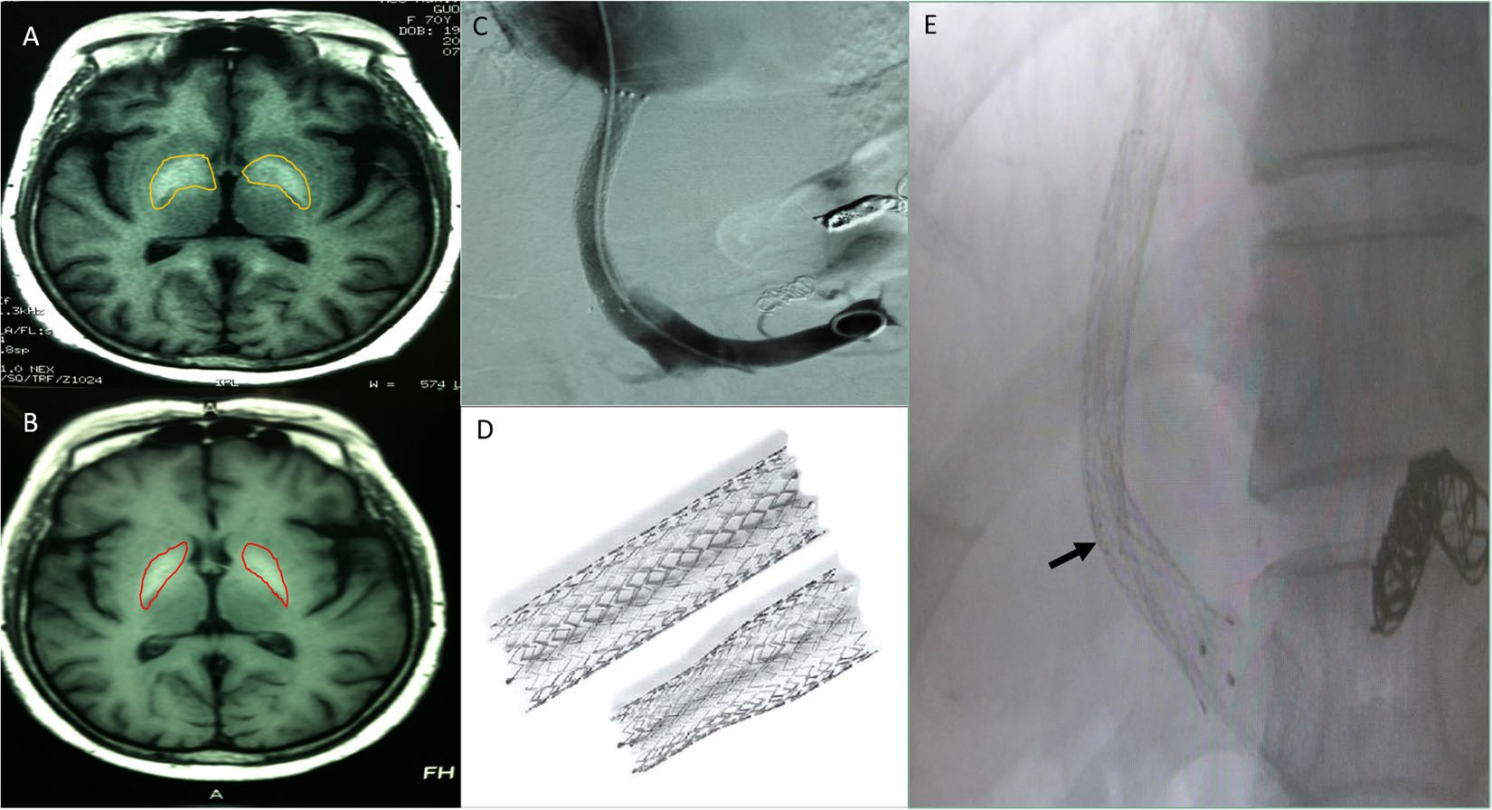
TIPS shunt limiting and cranial MRI changes. (**A**) Before stent limiting therapy, the cranial MRI T1WI exhibited high signals in the globus pallidus of the basal ganglia (yellow area). (**B**) After local stent-limiting about three months, the cranial T1WI MRI identified no aggravation and a slight decrease in the signals of basal ganglia (red area). (**C**) Angiography of portal vein after TIPS showed unobstructed stent and no abnormal varicose vein. The diameter of original shunt was 10 mm. (**D**) Normal straight stent and tapered stent image (below is 7–10 × 40 mm, ev3 Protégé Rx taper stent). (**E**) Patients underwent placement of a 7–10 mm (diameter) stent inside the original 10 mm stent. The distal point of stent was highlighted with arrows.

Post-treatment cranial MR imaging in 11 patients from group A exhibited slight reversal of previously identified demyelinated areas within basal ganglia and globus pallidus (Fig. 2A and B).

### Complications

In the course of this study, we focused on changes in perioperative conditions of patients subjected to stent-limiting procedures. The 17 (100%) shunt-limiting surgeries were successfully performed, without serious complications. In group A, one patient experienced severely low platelets (12 × 10^9^/L; normal reference range: 100–300 × 10^9^/L) caused by stress gastrointestinal bleeding on the day following surgery. The bleeding was controlled by platelet transfusions (2 units). The patient’s ADL/physical exercise capacities were improved during follow-up. Another patient developed hematemesis 3 months postoperatively (total volume: ~1000 ml). Emergency endoscopy revealed bleeding in gastric ulcer; and the patient recovered well after endoscopic treatment. Three patients developed refractory ascites symptoms (similar to pre-TIPS), which were controlled by diuretics. Two additional patients underwent LT.

In group B, three patients had melena several times after shunt-limiting treatments (each time bleeding ~100–150 ml). Endoscopy revealed esophageal variceal bleeding in two instances, and gastric ulcer bleeding in one case. The symptoms were improved after endoscopic hemostasis. Five patients developed refractory ascites (similar to pre-TIPS), which were controlled by diuretics in three instances. The other two required assisted abdominal punctures. One patient died from acute upper gastrointestinal bleeding 10 months after stent-limiting surgery, and one underwent LT.

### Overall survival (OS)

**Figure 3:**
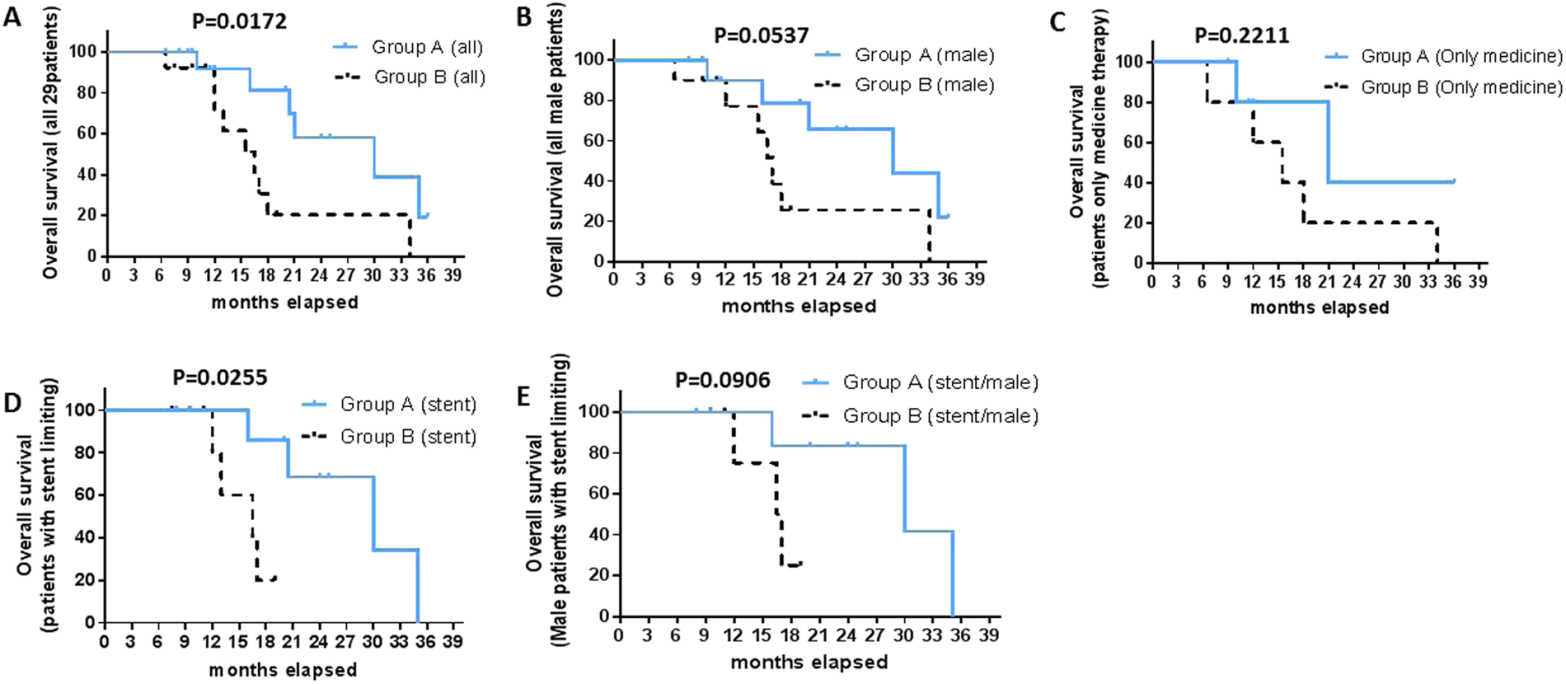
Estimated overall survival in group A and group B (Kaplan Meier Curves and log-rank test). (**A**) The median survival time of Groups A (30 months) was longer than Group B (16.5 months) (p = 0.0172). (**B**) The median survival time of male patients in Group A (30 months) was borderline longer than Group B (17 months) (P = 0.0537). (**C**) The median survival time of patients who only received medicine therapy in Groups A (21 months) was comparable to Group B (15.5 months) (P = 0.2211). (**D**) The median survival time of patients with stent limiting in group A (30 months) was longer than group B (16.5 months) (P = 0.0255). (**E**) The median survival time of male patients with stent limiting in group A (30 months) was borderline longer than group B (16.75 months) (P = 0.0906).

By log-rank (Mantel-Cox) test, median OS in group A was significantly different from group B (30 months vs. 16.5 months; log-rank test p = 0.017; hazard ratio [HR]: 0.322, 95% CI: 0.109–0.948), whereas borderline significantly different among male patients in group A from group B (30 months vs. 17 months; p = 0.054; HR: 0.355, 95% CI: 0.107–1.181). The median OS was comparable among patients given medical therapy in groups A and B (21 months vs. 15.5 months; p = 0.221; HR = 0.379, 95% CI 0.086–1.667). However, stent-limiting treatment recipients in groups A had prolonged survival time than group B (30 months vs. 16.5 months; p = 0.025; HR = 0.2626, 95% CI: 0.048–1.448). The median OS in male recipients of stent-limiting treatment in groups A was better than group B with borderline significance (30 months vs 16.75 months; p = 0.091; HR: 0.303, 95% CI: 0.046–2.013) (Fig. 3).

### Changes in portosystemic pressure gradient (PSG) and hepatic venous pressure gradient (HVPG)

In group A, significant increases in PSG were recorded from 16 patients (from 8.63 ± 0.89 to 9.63 ± 1.46 mmHg; t = 4.472; *p* = 0.0004); especially in male patients (from 8.58 ± 0.9 to 9.5 ± 1.24 mmHg; t = 4.005; *p* = 0.0021); and in stent-limiting treatment recipients (from 9 ± 0.87 to 10.44 ± 1.33 mmHg; t = 4.914; *p* = 0.0012). HVPG was increased significantly in patients receiving medical therapy (from 8.79 ± 0.49 to 9.29 ± 0.39 mmHg; t = 4.583; *p* = 0.0038). In group B, significant changes in PSG were recorded from 13 patients (from 8.39 ± 0.65 to 9.46 ± 1.2 mmHg; t = 3.482; *p* = 0.0045); especially in male patients (from 8.4 ± 0.7 to 9.6 ± 1.08 mmHg; t = 4.811; *p* = 0.001); and in stent-limiting treatment recipients (from 8.38 ± 0.74 to 9.25 ± 1.17 mmHg; t = 2.497; *p* = 0.0412) but not in medical therapy recipients (from 8.4 ± 0.55 to 9.8 ± 1.3 mmHg; t = 2.333; *p* = 0.08). On the other hand, all patients displayed significant increases in HVPG (from 8.39 ± 0.85 to 8.96 ± 0.32 mmHg; t = 2.232; *p* = 0.0455).

### Physical strength and function evaluation

**Figure 4:**
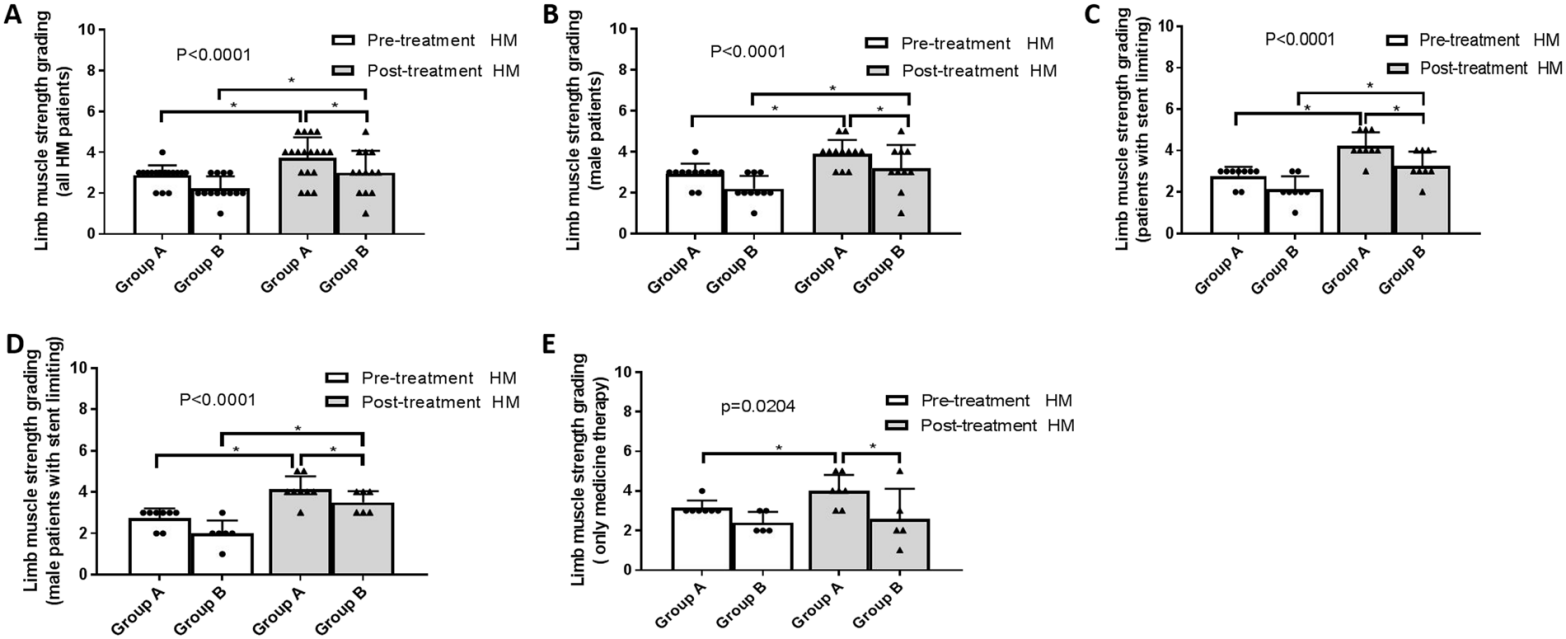
Lower limbs muscle strength grading pre- and post-therapy. (**A**) The muscle strength was improved more obviously in group A (t = 3.22, p = 0.005) than group B (t = 2.245, p = 0.0444) (F = 8.419, p < 0.0001). (**B**) The muscle strength was improved more efficiently among male patients from group A (t = 3.633, p = 0.0039) than group B (t = 2.535, p = 0.0319) (F = 9.611, p < 0.0001). (**C**) The muscle strength was more profoundly improved among patients with stent limiting from group A (t = 5.965, p = 0.0003) than group B (t = 2.826, p = 0.0256) (F = 17.45, p < 0.0001). (**D**) The muscle strength was improved better among male patients with stent limiting from group A (t = 5.227, p = 0.0012) than group B (t = 4.392, p = 0.0071) (F = 17.83, p < 0.0001). (**E**) The muscle strength among patients with medical therapy only was borderline improved in group A (t = 2.121, p = 0.0781) but not group B (t = 0.343, p = 0.7489) (F = 4.093, p = 0.0204).

**Figure 5:**
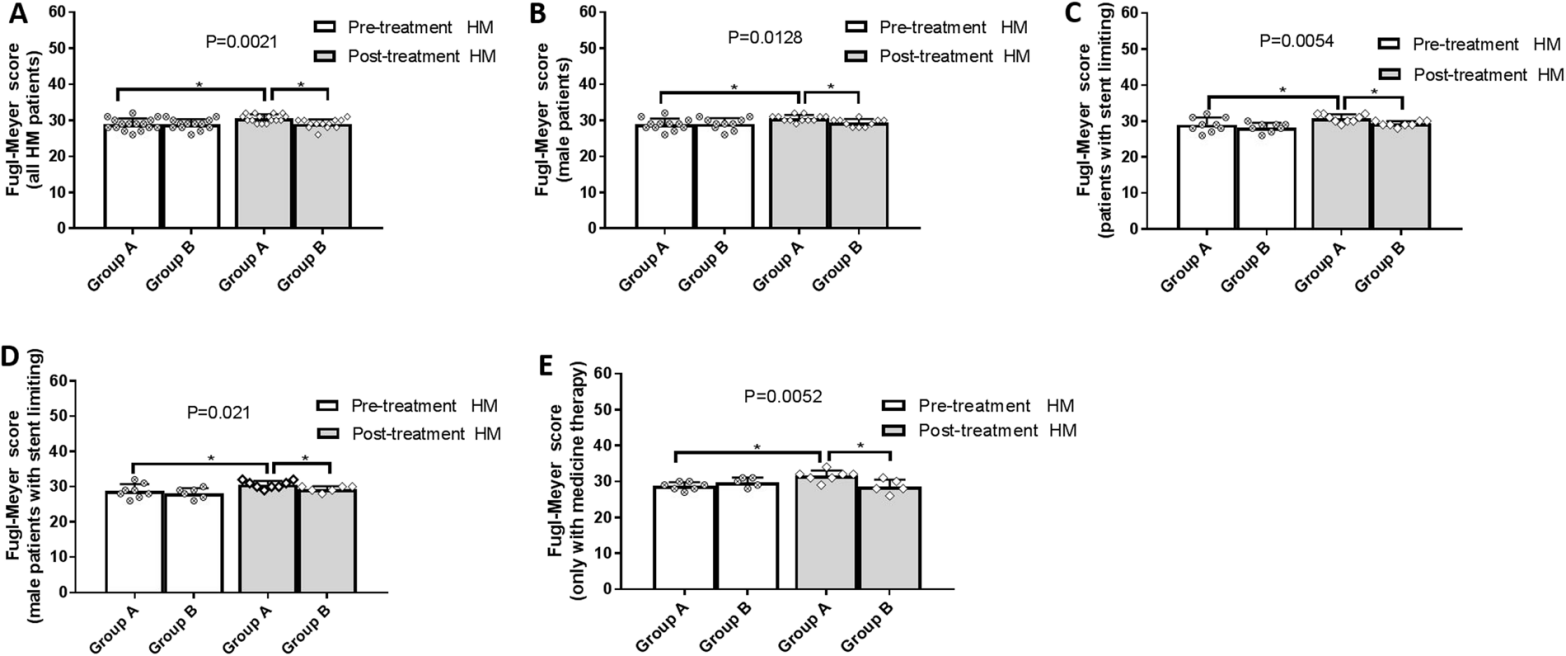
Lower limbs Fugl-Meyer scores pre- and post-therapy. (**A**) Fugl-Meyer score was improved in group A (t = 4.818, p = 0.0002) but not group B (t = 0.2976, p = 0.7711) (F = 5.575, p = 0.0021). (**B**) Fugl-Meyer score was improved among male patients in group A (t = 3.783, p = 0.0003) but not group B (t = 0.4737, p = 0.647) (F = 4.077, p = 0.0128). (**C**) Fugl-Meyer score among patents with stent limiting was improved in group A (t = 3.108, p = 0.0145) but not group B (t = 1.764, p = 0.1211) (F = 5.165, p = 0.0054). (**D**) Fugl-Meyer score was improved among male patients with stent limiting in group A (t = 3.934, p = 0.0219) but not group B (t = 1.557, p = 0.1801) (F = 3.903, p = 0.021). (**E**) Fugl-Meyer score was improved among patients with medical therapy only in group A (t = 8.402, p = 0.0002) but not in group B (t = 1.809, p = 0.1447) (F = 5.773, p = 0.0052).

**Figure 6:**
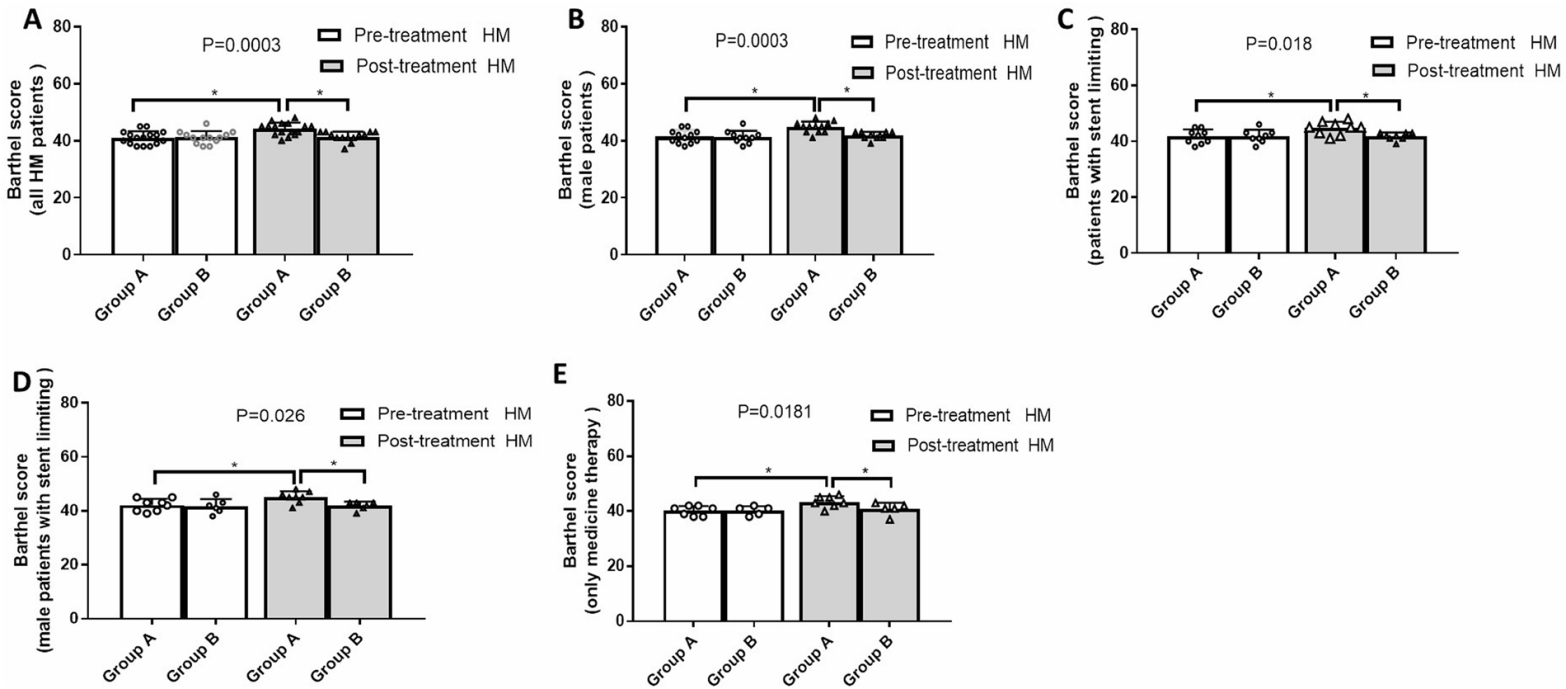
Lower limbs Barthel scores pre- and post-therapy. (**A**) Barthel score was improved in group A (t = 4.424, p = 0.0005) but not group B (t = 0.3712, p = 0.717) (F = 7.421, p = 0.0003). (**B**) Barthel score was improved among male patients in group A (t = 4.493, p = 0.0009) but not group B (t = 0.6547, p = 0.5291) (F = 7.755, p = 0.0003). (**C**) Barthel score was improved among patients with stent limiting in group A (t = 3.402, p = 0.0093) but not group B (t = 0, p > 0.999) (F = 3.912, p = 0.018). (**D**) Barthel score was improved among male patients with stent limiting in group A (t = 2.904, p = 0.0228) but not group B (t = 0.134, p = 0.899) (F = 3.68, p = 0.026). (**E**) Barthel score was improved among patients with medicine therapy in group A (t = 2.674, p = 0.0368) but not group B (t = 1, p = 0.3739) (F = 4.23, p = 0.0181).

Patients in group A had experienced significant improvement than group B in terms of lower-limb muscle strength, physical activity, and Barthel ADL index. All patients in group A routinely exhibited improved lower-limb muscle strength, physical activity, and Barthel scores, especially among male patients and those undergoing stent-limiting procedures. In group B, male patients and those receiving stent-limiting treatments obtained significant improvement on muscle strength. Before and after treatment, Lower-limb Fugl-Meyer and Barthel scores were similar in group B (Figs 4, 5 and 6).

## Discussion

TIPS is a highly efficient means of treating gastrointestinal hemorrhage and refractory ascites due to portal hypertension. Indications for TIPS, including first-line treatment recommendations, have been compiled by de Franchis and Baveno VI^8^. General awareness of post-TIPS complications has grown with worldwide application of TIPS. Complications of shunt stenosis or occlusion have been dramatically reduced by covered stents^9,10^. Symptoms of hepatic encephalopathy may be readily alleviated through ammonia-reducing drugs and restriction on oral protein intake.

However, special instances of TIPS-related HM presenting as rare forms of progressive spastic paraparesis have come under clinical scrutiny in recent years^10–12^. Although the pathogenesis of HM is not yet understood, extensive portosystemic shunting is clearly implicated. Possibly, circulatory shunting allows byproducts of nitrogenous degradation or a potential neurotoxin to escape hepatic detoxification, causing damage to the spinal cord. Interestingly, Nardone^13^ found that motor evoked potentials (MEP) in patients with more advanced disease were not substantially altered by LT, whereas LT conferred notable neurophysiologic and clinical benefit in patients with early stages of HM. Such findings suggest that HM may be reversible if treated early and timely, perhaps avoiding liver transplantation.

In our study, men (22/29, 75.86%) accounted for the preponderance of patients with HM. PSG was rising in both groups (group A: 12/16, 75%; group B: 10/13, 76.92%), especially in male recipients of stent-limiting treatment (group A: 8/9, 88.89%; group B: 6/8, 75%). However, HVPG was comparable in group A and B due to various confounders (e.g., respiratory rate, anesthesia). We presumed that changes in HVPG should parallel to PSG after stent-limiting procedures. Any upper gastrointestinal bleeding and ascites developing after stent-limiting treatment may therefore be attributed to an increase in PSG. All such symptoms experienced by our patients were ultimately remedied without serious or fatal complications. In our department, super-selective embolization is achieved through controllable Coil (Cook Medical) and Interlock (Boston Scientific); according to Wang^14^, Amplatzer vascular plugs (Abbott Laboratories, Chicago, IL, USA) may be used in lieu of coil to occlude large surgically implemented splenorenal shunts.

The muscle strength and activity of the lower limbs of group A were improved compared with those of group B, so the patients of group A accepted the better self-care ability and insisted on outpatient follow-up. However, there were three patients died of lung infection and 1 patient died of heart failure induced by long-term bed in group B. The mortality of group B (4, 30.77%) is greater than group A (2,12.5%). Anymore 2 patients (group B) lost contact in follow up. Therefore, patients in the group A have significantly longer estimated OS than those in the group B. As mentioned above, a majority of patients were male. A significant difference in OS between group A and B was observed, whereas a border-line difference in OS in male patients or male stent-limiting treatment recipients. Because only three female patients (one in group A; two in group B) underwent stent-limiting treatment, statistical analysis was not performed. A larger sample size is guaranteed to explore if stent-limiting treatment can conceivably impact outcomes of female recipients in both groups. The OS of patients receiving medical therapy was comparable by group.

The muscle strength assessment indicated significant improvement in overall patients. There were four male patients in each group (group A: 4/7, 57.14%; group B: 4/5, 80%) who received medical therapy only. Fundamentally, muscle strength represents function of muscle fibers and cells. Although men generally surpass women in this regard, their recovery from regional impairment can be slowed. Men are typically less compliant than women in the course of medical treatment. Fugl-Meyer and Barthel scores are gauges of lower-limb activity and ADL capacity. Only patients of group A showed obvious improvement in these two closely related indices.

In our study, 17 patients with HM secondary to TIPS underwent shunt-limiting surgery. By reducing internal shunt diameters (and thus portosystemic flow fractions), blood volumes directly entering systemic circulation without hepatic detoxification are lowered, limiting injurious spinal cord exposures. In our previous study^7^, patients with HM who undergo TIPS-limiting surgery recovered in lower-limb muscle strength 3–6 months thereafter, with significant improvement in exercise and ADL capacities. At 6–12 months postoperatively, these parameters were partially improved, with no clinical symptom aggravation. However, subjective influences are of relatively minor importance. Lower-limb exercise capacity remains progressive decline as cirrhosis progresses and muscle strength is lost. Abnormal signals in areas of the basal ganglia and globus pallidus were changed as clinical symptoms abated, consistent with descriptions by Chavarria and Wang^15,16^. Conn^17^ performed stent blockage in one patient with HM following TIPS, which improved symptoms, emphasizing positive effects of timely shunt closure. Only one of our patients (in group B) suffered from a poor postoperative outcome, despite successful shunt-limiting surgery, succumbing to acute gastrointestinal bleeding and deteriorating liver function. It should be noted that portal vein pressures were rising after shunt-limiting surgery (compared with baseline values), therefore, complete TIPS occlusion was not recommended. Imaging studies of the portal system should also be perfomred after shunt-limiting procedures; and if aberrant varicosities ensue, good care should be taken to prevent new deadly bleeding.

The present study has several limitations. Its retrospective design inherently is prone to selection bias. Furthermore, our data were locally restricted, drawn from patients inhabiting in Northern China. Larger controlled clinical trials are needed to corroborate these results. We believe this pilot study will enhance our knowledge about evaluation and management of patients with HM. To date, this is the largest multicenter study to assess the effects of therapy on limb activity in the context of post-TIPS HM.

In summary, although median survival times in male recipients of HM treatment were independent of symptomology, early stent-limiting surgery significantly improved postoperative outcome. We strongly advocate active therapeutic intervention as soon as feasible once HM is confirmed after TIPS, especially within 6 months of symptoms onset. By adding stent-limiting surgery to medical therapy, lower-limb muscle strength can be improved significantly in these patients; and treatment administered within 6 months rather than later promote better lower-limb activity and ADL capacity by comparison.

## Materials and Methods

### Clinical data

We collected demographic and clinical information on 3467 patients undergoing TIPS at three Hepatology Centers of Northern China’s largest Liver Disease Hospitals (Beijing Shijitan Hospital, Beijing Ditan Hospital, affiliate Hospital of Capital Medical University; and 302 Military Hospital of China) between August 1994 and May 2016. This study complied to ethical standards of institutional research committees, abiding by the 1977 Declaration of Helsinki and its later amendments or comparable ethical standards. The study was conducted according to the Standards of Practice Guidelines of the Cardiovascular and Interventional Radiological Society of China and approved by the ethics committee/institutional review board of above 3 Hospitals (the ethics committee of Beijing Shijitan Hospital, the ethics committee of Beijing Ditan Hospital, the institutional review board of 302 Military Hospital of China). Informed consent was obtained from all individual participants or their relatives included in the study. The patients and their relatives were informed about the potential outcomes and risks of TIPS procedure. A written consent to undergo TIPS and to participate in this research was signed by each subject. This manuscript contains no information or images that could lead to identification of a study participant.

Diagnostic criteria of HM included^18–21^: (1) a history of chronic liver disease and underwent TIPS treatment; (2) progressive spastic paraplegia, without obvious sphincter and shallow sensory dysfunction; (3) no symptom of spastic paraplegia before TIPS; and (4) normal cerebrospinal fluid and serum blue copper protein, no Kayser-Fleischer ring. Exclusion criteria of HM included^18–21^: (1) spinal cord lesions caused by spinal cord space-occupying mass, multiple sclerosis, hepatolenticular degeneration, infectious, inflammatory, autoimmune, vascular, hereditary, and metabolic causes; (2) severe hepatorenal damage; (3) severe infection and systemic cachexia; (4) serious cardiovascular diseases; and (5) cardiac portal hypertension.

### Study design

Patients were diagnosed with myelopathy and corticospinal tract damage after joint consultation (radiologist, physician, and neurologists). Each diagnosis of HM was based on available diagnostic criteria, after excluding other causes of myelopathy^20,21^. we detected symptoms of HM within 1.5–26 months after TIPS; the time from consultation to treatment was ranged from 1.5–38 months. In the previous study^19^, for patients with symptoms of HM for <3 months, clinical efficacy was achieved through conservative medicine; for patients with symptoms of HM for 3–6 months, conservative medical treatment provided clinical efficacy in few of them, whereas others did not respond similarly, requiring combined shunt-limiting surgery for improvement. Patients were thus grouped according to the time of onset of HM symptoms. Because all patients initially experienced symmetric limping gait of both lower limbs, the time-lapse from lower-limb symptom onset to start of clinic treatment was used to stratify patients into group A (<6 months) and group B (≥6 months).

### Conservative medical treatment

Twelve of the 29 patients (group A, 7; group B, 5) received conservative medical treatment, using the following regimens: (1) oral protein restriction (<20 g/day); (2) oral lactulose administration (three times daily); (3) oral vitamin B1 (20 mg three times daily); (4) intramuscular mecobalamin (1 mg once daily); (5) oxygen-free radical scavenger (edaravone, 3 0 mg twice daily for at least 4–6 weeks); and (6) physical therapy (at least 8–12 weeks of physiotherapy and rehabilitation training).

### Original TIPS procedures

Shunts between hepatic vein, inferior vena cava, and portal vein were established. After topical disinfection and local anesthesia (1% lidocaine) injection, internal jugular vein was punctured, and an angiographic catheter was inserted to delineate hepatic vein and inferior vena cava. Hepatic vein or hepatic segment of inferior vena cava was then accessed (RUPS-100 apparatus; Cook Medical Inc, Bloomington, IN, USA); subsequently an intrahepatic portal branch was punctured at an appropriate position and angle for setting placement within portal vein. Based on portography and portal venous pressure (measured via catheter), varicosities were embolized using plugs and/or gelatin sponge before or after shunt establishment, and portal venous pressure was again measured. Angioplasty balloons (diameters of 8 and 10 mm, respectively) were successively inserted via guide wire to dilate the flow passage, and a stent (bare, eV3 Protégé [Medtronic, Minneapolis, MN, USA], Cordis SMART [Cardinal Health, Dublin, OH, USA], or Fluency covered [Bard Peripheral Vascular, Tempe, AZ, USA]) of 8- or 10-mm size was placed, followed by portal venous and systemic pressure gradient (PSG) measurement and portography (Fig. 2C).

### TIPS shunt-limiting

Seventeen patients (group A, 9; group B, 8) underwent shunt-limiting procedures through classic TIPS approach. Stents of 10-mm diameter (7–10 × 40 mm ev3 Protégé Rx tapered stent or 10 × 80 mm eV3 stent; Medtronic) or 8-mm diameter (6–8 × 40 mm eV3 Protégé Rx tapered stent; Medtronic) were placed within the existing stents (Fig. 2D and E). Digital subtraction angiography (Siemens AXIOM Artis dTA; Siemens, Munich, Germany) and abdominal vascular ultrasound (LOGIQ system; GE Healthcare, Chicago, IL, USA) were performed to gauge inner shunt diameters. After implantation of the limiting stent, no visible stent shift was visible, and the shunt was completely opened at both ends. No appearance of new varicose veins suggested successful shunt-limiting.

### Follow-up and evaluation indices

Patients were monitored for 1–36 months (mean, 16.74 ± 8.61 months) after admission for therapy, the primary clinical endpoint being all-cause mortality. Secondary endpoints were shunt dysfunction (defined as shunt occlusion) and clinical relapse (defined as recurrence of clinically significant bleeding or ascites requiring repeatedly paracentesis).

All subsequent complications were recorded, including any gastrointestinal bleeding, ascites, or portal vein thrombosis. During clinical visits, routine hematologic, biochemical, and coagulation profiles were obtained, in addition to blood ammonia levels. Changes in portosystemic pressure gradient (PSG) and hepatic venous pressure gradient (HVPG) before and after shunt-limiting procedures were recorded. The overall survival (OS) was calculated from the date of diagnosis to the date of last follow-up or death. Prior to and after treatment, patients were examined by two neurologists who conducted simple Fugl-Meyer assessment (FMA) of lower limb activity (34-point total score). Lovett’s six-point classification was likewise used to assess muscle strength, and the Barthel activities of daily living (ADL) index (100-point total score) served to measure patient disability. If the two-party scoring of patients differed, mean scores were applied. For the patients incapable of clinic visits, symptoms and complications were monitored through telephone interviews.

### Statistical analysis

Normal continuous variables were compared by Student’s *t*-tests. Pre- and post-treatment comparisons of liver function indices and PSG determinates were achieved by paired *t*-tests. Cumulative OS was estimated from Kaplan-Meier curves and compared by log-rank test. Univariate and multivariate analyses of time to event outcomes predictors were performed using the Cox proportional hazard regression models. A two-tailed *p-*value < 0.05 was considered statistically significant. All computations were performed with standard software (SPSS version 20.0 [IBM, Armonk, NY, USA] and GraphPad Prism 7 [GraphPad Software Inc, La Jolla, CA, USA]).

### Ethics approval

This study was approved by the China National Committee for Data Protection and by the local ethics committee at each participating center.

### Informed consent

All patients participating in this study signed an informed consent form.
